# Long-Term Improvement in Urinary Incontinence in an Elite Female Athlete Through the Laser Treatment: A Case Report

**DOI:** 10.7759/cureus.36730

**Published:** 2023-03-27

**Authors:** Nobuo Okui, Tadashi Ikegami, Aleksandra Novakov Mikic, Machiko Okui, Adrian Gaspar

**Affiliations:** 1 Dentistry, Kanagawa Dental University, Kanagawa, JPN; 2 Diagnostic Imaging, Kanagawa Dental University, Kanagawa, JPN; 3 Obstetrics and Gynecology, Polyclinic “Novakov I sar”, Novi Sad, SRB; 4 Urogynecology, Yokosuka Urogynecology Clinic, Kanagawa, JPN; 5 Urogynecology, Uroclinica Faculty of Medicine, University of Mendoza, Mendoza, ARG

**Keywords:** pelvic floor muscle training, anteroposterior and transverse urethral diameters, pelvic floor muscles, puborectalis muscle, therapeutic targets, erbium yag laser, elite female athletes, stress urinary incontinence

## Abstract

Stress urinary incontinence (SUI) is increasing in elite female athletes (EFAs), affecting competition results and quality of life. Pelvic floor muscle training (PFMT) is the first-line treatment for SUI, and surgery is generally performed when PFMT is insufficient. However, in EFA, there are few cases in which surgery is performed and fewer reports. Therefore, there is no known general treatment strategy for EFA with SUI.

In our study, a 23-year-old track-and-field medalist with severe SUI was successfully treated with a vaginal and urethral erbium-doped yttrium aluminum garnet laser (VEL + UEL). After 12 treatments over one year, urinary incontinence decreased from 300 mL or more in the 400 m track run before treatment to 0 mL. She did not experience any more problems during running or competition. There was no recurrence of SUI for three years, and the urethral pressure profile examination confirmed improvement.

MRIs showed that the left puborectalis muscle was absent from the first visit. The urethra was oval with an anteroposterior outer diameter of 10 mm and a transverse outer diameter of 13 mm before treatment. However, after three years of treatment, both anteroposterior and transverse diameters became circular, measuring 11 mm. Vaginal wall thickness increased from 8 to 12 mm at the center of the height of the urethra, making it possible to support the urethra, and pretreated adipose tissue space between the urethra and vagina disappeared.

It was noted that the uneven and fragile urethra/vagina, the presence of adipose tissue space, and the absence of the left puborectalis muscle may have been the cause of the SUI. One year of VEL + UEL treatment resulted in long-term improvement of SUI; MRI showed changes in the urethra and vagina.

## Introduction

Stress urinary incontinence (SUI) is urinary incontinence that occurs during abdominal pressure, such as coughing, exercising, and sneezing. It is often observed in postpartum women, with two to four times higher frequency in women than in men [1].

Although SUI is multifactorial, it is primarily caused by anatomical problems in the pelvic floor muscles (PFM) and urethra. In recent years, an increasing prevalence of SUI has been identified among young elite female athletes (EFAs) [2]; the prevalence of urinary incontinence (UI) in EFAs is approximately 25.9%, with SUI being the most common symptom [3]. It has been reported that 39% of EFAs at the international championship level consider SUI to impact negatively competition results and everyday life [4].

Management of EFA patients with SUI includes limiting physical activity, using tampons, and limiting fluid intake. However, these measures adversely affect their running form and physical conditions [4,5]. The first-line treatment is PFM training (PFMT) [6,7]. Subsequently, surgery is performed in cases that do not improve after PFMT [8]. SUI surgery includes urethral collagen injection and urethral sling surgery. For urethral injection, drugs are not available in some countries. In the case of urethral sling surgery, one might think that the surgical wound would affect running form, as in the case of the athlete in this case report.

In a retrospective study of adolescent females with SUI (mean age 15 years), 18 of the 33 subjects were involved in strenuous physical activity (hip/hop dancing, competitive gymnastics, and typical sports such as tennis and running). In this study, standing cystography showed bladder neck ptosis as a cause of SUI in 20 subjects. Fifteen of the 20 with bladder neck ptosis exercised vigorously, and three of the 13 without bladder neck ptosis exercised vigorously. Of the 20 in the group with bladder ptosis, three were lost to follow-up, six improved without surgery, and eight were managed with surgery (Burch procedure; five, fascial sling; two, urethral injection; one, and artificial urinary sphincter: one). The authors conclude that nonpregnant women with SUI who are active in sports should be considered for nonsurgical treatment, but will likely require bladder neck elevation. The authors also wrote that nonsurgical treatment is effective in female patients with minimal bladder descent on cystogram [8].

In recent years, an erbium-doped yttrium aluminum garnet (Er: YAG) laser treatment has been used for SUI women [9]. The mechanism of action of laser therapy for SUI is to increase temperatures in the mucosa and submucosa to 45-60°C through photo-thermal effect and reach (through heat dissipation) depths up to 400 µm beneath the mucosa surface. This effect promotes collagen remodeling, de novo collagen synthesis, and neoangiogenesis [9]. This effect may increase PFM support by altering the thickness of the urethra and vagina [9].

There are two methods for laser irradiation. One is the vaginal erbium SMOOTH laser (VEL) treatment [10], in which a special glass case is inserted into the vagina. A handpiece is inserted to irradiate the anterior wall and the entire vagina repeatedly. The second is intraurethral SMOOTH Er: YAG Laser (UEL) treatment. A specially processed thin metal tube handpiece is inserted into the urethra, and the target area is irradiated from the inside of the urethra [10].

A recent study shows a change in treatment choices regarding SUI among women who wish to become pregnant and have a baby again. In a propensity score matching study of 327 women treated for SUI, the authors found that while transvaginal tape (TVT) surgery, a type of prosthetic surgery, was previously the procedure of choice for women who wanted to conceive, they are now more concerned about prostheses and are more likely to choose PFMT or VEL. One of the reasons is that they are now more concerned about complications of prostheses [11].

There is no consensus on how mid-urethral slinging (MUS), including TVT, affects pregnancy and delivery. In modern times, some clinicians recommend that women postpone MUS surgery if they consider further pregnancies. Alternatively, cesarean section is routinely suggested as the method of delivery after MUS surgery, and some argue that future pregnancies need not be viewed as an absolute contraindication to MUS surgery since pregnancy after MUS did not increase the odds of SUI repeat procedures or return visits [12]. All of this makes it necessary to make a multifactorial decision to choose whether to use PFMT, MUS, or VEL in the case of EFAs who wish to conceive their next pregnancy.

In our study, we treated a case of urinary incontinence in an EFA who was a track-and-field medalist. This patient had SUI since she was a young athlete (under the age of 18 years), and it worsened after giving birth at the age of 22. Her SUI continued to deteriorate despite PFMT, so she ceased to compete. Concomitant VEL and UEL treatment (VEL + UEL) improved her urinary incontinence and competition results, enabling her to return to competing. This report is invaluable for understanding the mechanism of SUI in EFAs and therapeutic targets.

## Case presentation

A 23-year-old track and field-top EFA presented with complaints of urinary incontinence during exercise for six years. (Hereafter, “this case” refers to this athlete.)

During childhood, she did not experience incontinence, even during running. Her first bout of urinary incontinence occurred at age 17, with approximately 100 mL of urinary incontinence in a 400 m run. She started working on PFMT with national team trainers when she was selected as a national player at the age of 18. At the age of 22, she delivered a 2,800 g baby at 38-week gestational age, with no severe perineal laceration. After a four-month postpartum rest, she resumed her practice. Since having a vaginal delivery, her urinary incontinence worsened to such an extent that she experienced more than 300 mL in a 400 m run. She alternated between resistance training and PFMT during eight hours of athletics practice under the guidance of a track and field athletic trainer. After six months, there was no improvement. After this, the PFMT continued throughout the entire process. Using pads and restricted fluid intake interfered with her competitive life, and urinary incontinence prevented her from continuing to compete.

On her first visit, she used six pads daily to control her urinary leakage. The patient had no pelvic disease or diseases that could have caused SUI. She had no history of smoking or drinking, genetic disorders, or previous surgery. She was not taking any medications. No recurring urinary or urinary tract abnormalities were observed. She had no history of the disease nor a family history of kidney or bladder diseases.

At her initial physical examination (referred to as T0), the International Consultation on Incontinence Questionnaire Short Form (ICIQ-SF), overactive bladder symptom score (OABSS), a one-hour pad test (1-h pad), 400-m sprint pad test (400 m pad), urodynamic study (UDS), urethral pressure profile (UPP), modified Oxford scale (MOS), and magnetic resonance imaging (MRI) was performed.

The ICIQ-SF is the questionnaire to assess the frequency, severity, and impact of urinary incontinence on quality of life in research and clinical practice. It is divided into the four categories: very severe (19-21), severe (13-18) moderate (6-12), and slight (1-5). The OABSS is a symptom assessment questionnaire which is designed to quantify overactive bladder symptoms. OABSS shows three categories: severe (over 12) moderate (6-11), and slight (under 5). The one-hour pad test measures the pad weight according to standard protocols. It is rated as mild (2-10 g), moderate (11-50 g), or severe (>50 g), depending on pad weight [13]. The clinical improvement level was defined as a more than 50% reduction in pad weight.

The 400 m pad is a test that continues a measurement protocol used to record data for this patient when she was a youth athlete. After the patient’s bladder filled to its maximum capacity with urine, the patient was asked to run at full speed around a 400-m track once, after which the pad’s weight was measured.

For PFMT, two physicians independently palpated the vagina. PFM contractions were categorized as follows: no visible contraction, opposite direction (tension manipulation or Valsalva), simultaneous contraction of accessory muscles, or correct PFM contraction. After palpation, the opinions of the two doctors were combined.

MOS was used to measure vaginal pressure. A doctor inserted two distal phalanges into the vaginal orifice and evaluated PFM contractions on the following six scales: 0, no contraction; 1, a flicker; 2, weak contraction; 3, moderate contraction; 4, good contraction; and 5, strong contraction. The Goby Family of Wireless Urodynamics Systems was used for the urodynamic testing and UPP (EDAP TMS SA, Rhône, France; air-filled dual-sensor catheter; retraction rate: 1 mm/s).

For MRI, the patient's pelvis was scanned using a 1.5-T MRI machine (GE Healthcare, Milwaukee, WI, USA). Sagittal section imaging conditions for T2-weighted images were TR4500, TE110, FA140°, Acquisition Matrix 320 × 320, NEX 2, 3 mm thickness, and 3.5 mm space using the propeller method. Horizontal section conditions were TR4500, TE100, FA140°, Acquisition Matrix 380×380, NEX1.4, 5 mm thickness, and 6 mm space. Imaging data were examined to quantify urethra and vagina size and to observe surrounding organs. Lesions were visually assessed and manually labeled by a trained physician.

The degree of interference with competition was assessed by the individual with a visual analog scale (VAS) score (range 0-10; 0 = absence of symptoms, 10 = worst symptoms). We specifically asked her thoughts on the competition.

The intervention methods were as follows: VEL, UEL, or concurrent treatment (VEL + UEL) because treatment with open surgery may affect competition results. She did not wish to go abroad for collagen injections into her bladder. This EFA, the track and field athletic support team, and we believed that PFMT alone, continued over a long period of time, would not improve urinary incontinence. VEL and UEL were found to be safe in 113,000 women data [14].

First, we disinfected the patient's vagina, labia, and urethra. An 8% xylocaine spray (Sandoz K.K., Tokyo, Japan) was applied. No sedatives were used. The VEL step used renovalase (SP Dynamis Fotona, Ljubljana, Slovenia) with a glass vaginal speculum and vaginal probe. The anterior vaginal wall was treated with a PS03 laser probe with 2.0 Hz frequency, 6 J/cm^2^ pulse fluence, and a 7 mm spot size. The treatment was repeated thrice, irradiating the target area every 5 mm.

Next, an R11 laser probe was used. The pulse covered 360 degrees of the vaginal canal with 2.0 Hz frequency, 7 mm spot size, and 3.00 J/cm^2^ pulse fluence. The vaginal canal was laser-irradiated every 5 mm, and the procedure was repeated twice. This process was completed in approximately 20 minutes [9].

The same laser model was used in the UEL step. We used an R09-2Gu laser probe engineered to enter the urethra after bladder emptying via catheterization. Laser therapy was performed at R09-2Gu, Smooth, 1.4 Hz, 1.5 J/cm^2^, and four stacks in 2.5 mm increments from the urethral opening to the proximal end. This treatment was repeated four times and completed in approximately 10 minutes [10].

VEL + UEL, VEL, and UEL were performed consecutively. The patient was advised to avoid sexual intercourse and masturbation for one week after treatment.

One month after the first VEL treatment (L1), no improvement in urinary incontinence during the competition and no change in competitive VAS score were observed. Next, UEL treatment was performed (L2). One month after L2, urinary incontinence decreased by 10%, while the VAS score remained unchanged. Subsequently, VEL + UEL treatment was administered (L3). One month after L3, urinary incontinence decreased by 20%, and the VAS score was 8 (decreased by two). Thereafter, VEL + UEL treatment was performed monthly (L4-L12): one, two, and three years after L12 are marked as T1, T2, and T3, respectively.

Because there was no precedent for whether laser urinary incontinence treatment could qualify as doping in the Olympics, after one year of laser treatment, the patient waited another year before returning to compete; she returned to track and field competitions at T2. After a year of match experience, we re-evaluated her at T3.

Pretreatment (T0) findings

**Figure 1:**
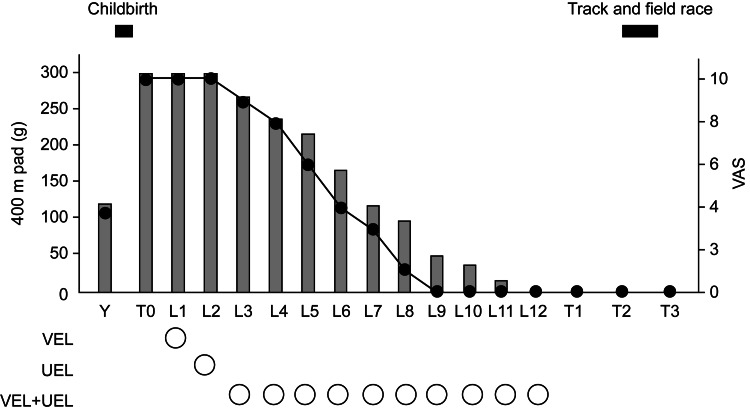
The 400 m pad test and the degree of interference with the competition Bar graph: 400 m pad: Weight of urinary leakage (g) of the pad during a 400 m track running with urine collected to the maximum capacity of the bladder Line graph with black circles: VAS: Degree of hindrance to competition (0: no hindrance to 10: maximum hindrance) Y: Youth Player Period, VAS: Visual analog scale, VEL: Vaginal erbium SMOOTH laser, UEL: Intraurethral SMOOTH Er: YAG Laser, VEL+UEL: Vaginal and urethral erbium-doped yttrium aluminum garnet laser, T0: Before treatment, L1 to L12: Laser treatment (performed monthly), T1 to T3: one to three years after completion of laser treatment Track and field race: Track and field competition held between T2 and T3

The 14th month of vaginal delivery. The ICIQ-SF shows 16 (severe). The OABSS is 0. The one-hour pad was moderate at 15g. The 400 m pad test showed more than 300 g, and most of the urine in the bladder leaked (Figure 1). UDS results showed normal bladder function and compliance. Detrusor contractility was low at 19 cmH_2_O. UPP results were lower than the standard: Valsalva leak point pressure (VLPP) was 66.8 cmH_2_O, maximum urethral pressure (MUP) was 55 cmH_2_O, and maximum urethral closure pressure (MUCP) was 54 cmH_2_O. (Average Japanese MUP: 74.3±21.6; MUCP: 66.4±15.8 [15]). A normal VLPP (≥60 cmH_2_O) and a normal MUCP (>20 cmH_2_O) rule out intrinsic sphincter deficiency (ISD) [16]. The final diagnosis was SUI.

PFM contractions were judged as correct contractions by two physicians and rated with a MOS of 4. When asked about her thoughts on athletics, she replied that this condition had been a burden since her time as a young athlete. Immediately prior to treatment, her VAS score was 10.

Figure 1 shows the amount of urinary incontinence and VAS during the 400 m race. As the number of laser treatments increased from L1 to L12, the 400 m pad volume decreased, and VAS was observed.

Post-treatment (T3) findings

Both the ICIQ-SF and the OABSS are 0. The 1-h pad was 0 g and urinary incontinence disappeared. The 400 m pad was 0 g and urinary incontinence disappeared (Fig. 1). PFM contractions were the same as those at T0 and judged as correct contractions. The MOS was 4. UPP results were 99 cmH_2_O for MUP and 78 cmH_2_O for MUCP.

The patient was returned to the athletic department. The VAS score disappeared completely at L9 and remained until T3. The patient no longer needed practice pads for three years after treatment. At L9, she explained that the discomfort of her pelvic organs from swinging around had disappeared, making it easier for her to run short distances.

Adverse effects

No adverse effects of the VEL + UEL treatment were observed during the entire treatment course.

MRI findings

The anteroposterior and transverse urethral diameters on MRI were measured at the center of the urethra, according to previous reports. The method for measuring urethral diameter followed those used in the same report [17]. The reported anteroposterior diameter was 15 ± 2 mm (range: 11-20 mm), and the transverse diameter was 15 ± 2 mm (range: 12-19 mm). Therefore, the shape was circular, front, rear, left, and right diameters were 15 mm or more, and the vertical diameter was 22 ± 3 mm (range: 17.6 to 26.4 mm); therefore, 20 mm or more is considered normal [17]. As shown below, the values, in this case, were lower than normal values both before and after treatment.

**Figure 2:**
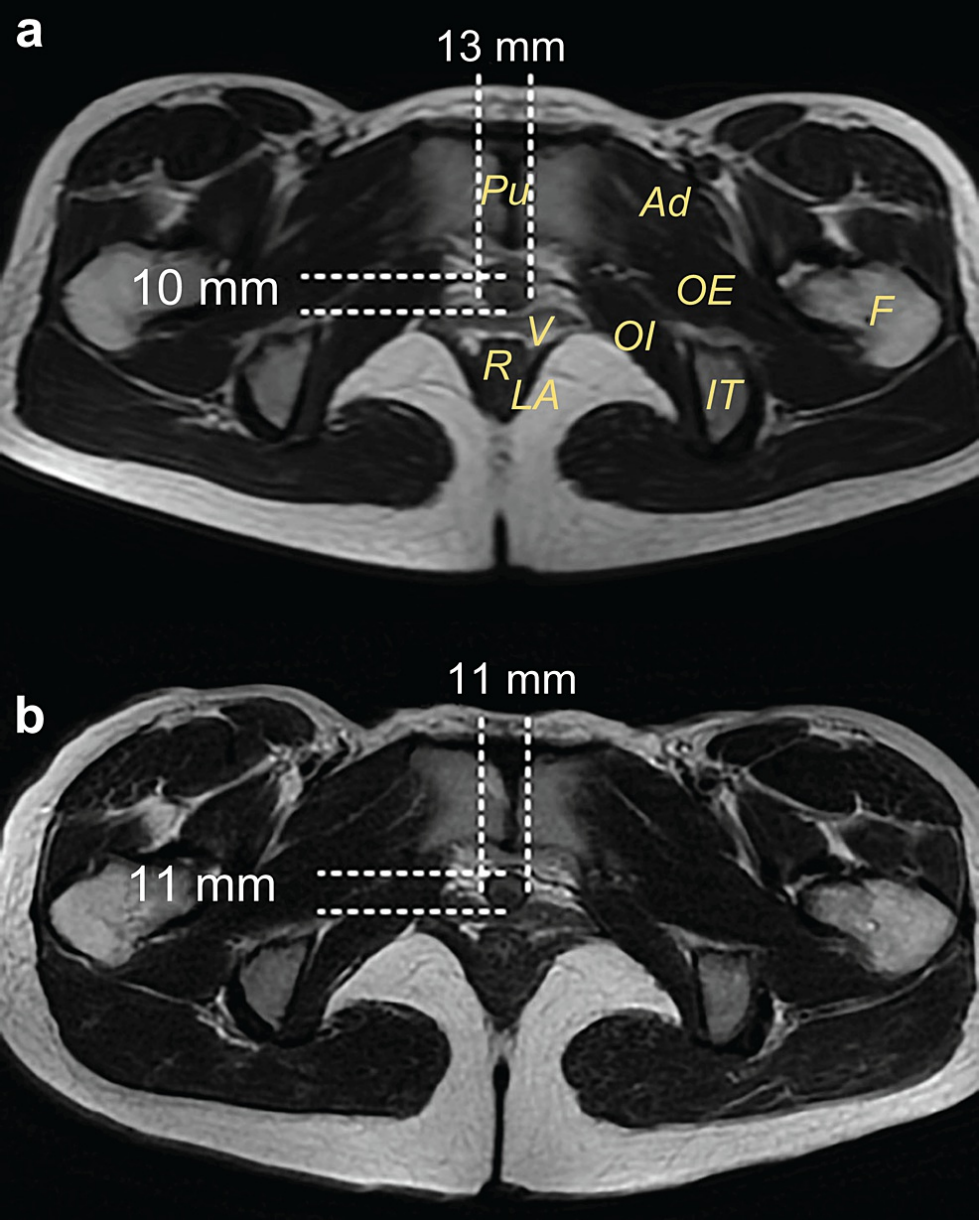
Morphology and size of the urethra at the level of the striated sphincter muscle in the axial view (a) Before treatment (T0), (b) After treatment (T3) T0: The transverse diameter 13 mm, anteroposterior diameter 10 mm T3: The transverse diameter 11 mm, anteroposterior diameter 11 mm Pu, pubic bone; V, vagina; R, rectum; Ad, adductor muscle; OE, obturator externus muscle; OI, obturator internus muscle; IT, sciatic tubercle; LA, Levator ani muscle; F, femur

Figures 2a, 2b show MRIs for this case at T0 and T3, respectively. The morphology of the urethra was elliptical at T0 and circular at T3. The anteroposterior urethral diameter at T0 was 10 mm, and the transverse diameter was 13 mm. At T3, they were both 11 mm, respectively.

**Figure 3:**
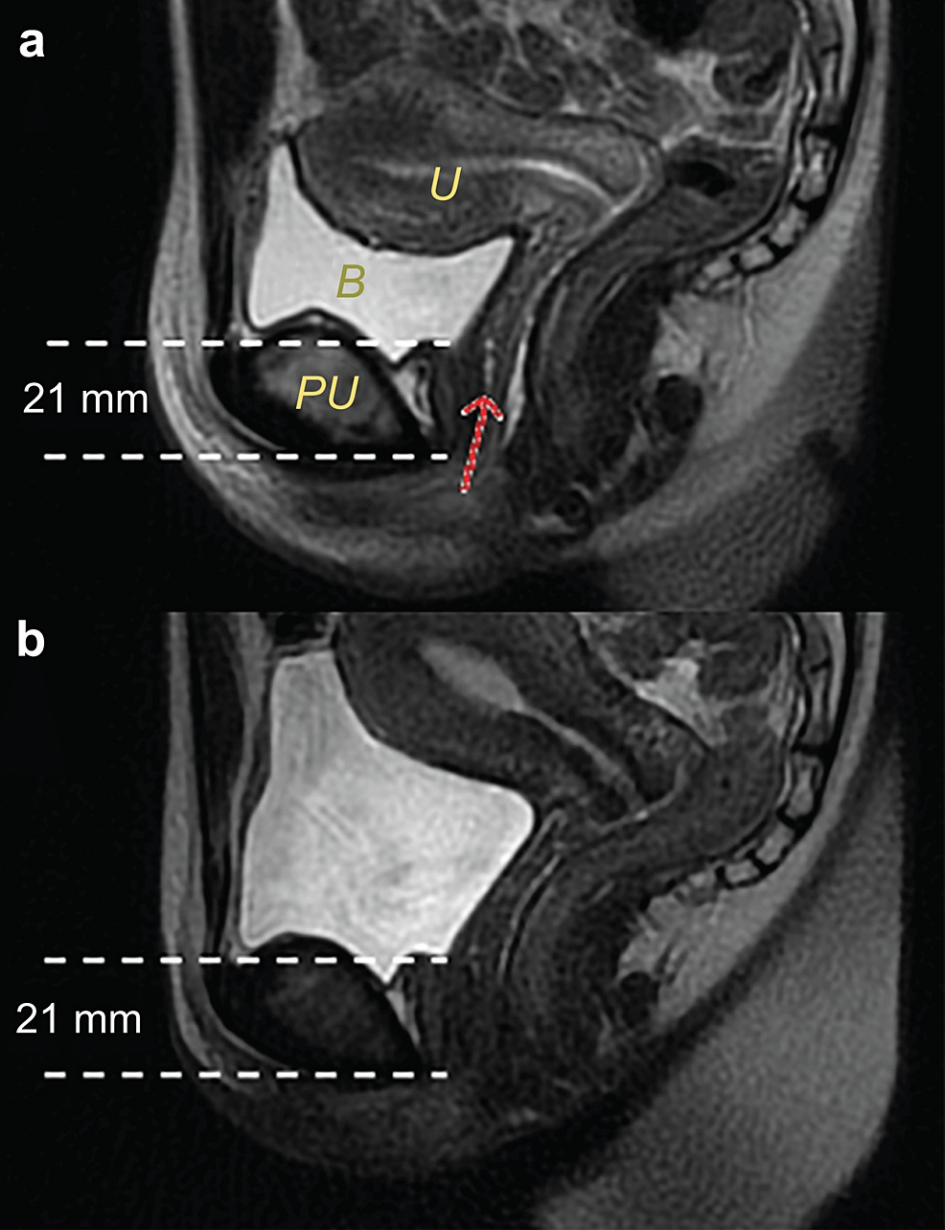
Median sagittal view showing the longitudinal urethral diameter and adipose tissue space a: Before treatment (T0) b: After treatment (T3) T0: Vertical urethral diameter 21 mm, T3: 21 mm U, uterus; B, bladder; Pu, pubic bone Red arrow: fat layer space

Figures 3a, 3b show the urethral longitudinal diameter and adipose tissue space surrounding the urethra and vagina, respectively. The urethral diameter was 21 mm at both T0 and T3. At T0, there was an adipose layer between the vagina, rectum, and urethra, as indicated by the arrows, which disappeared at T3.

**Figure 4:**
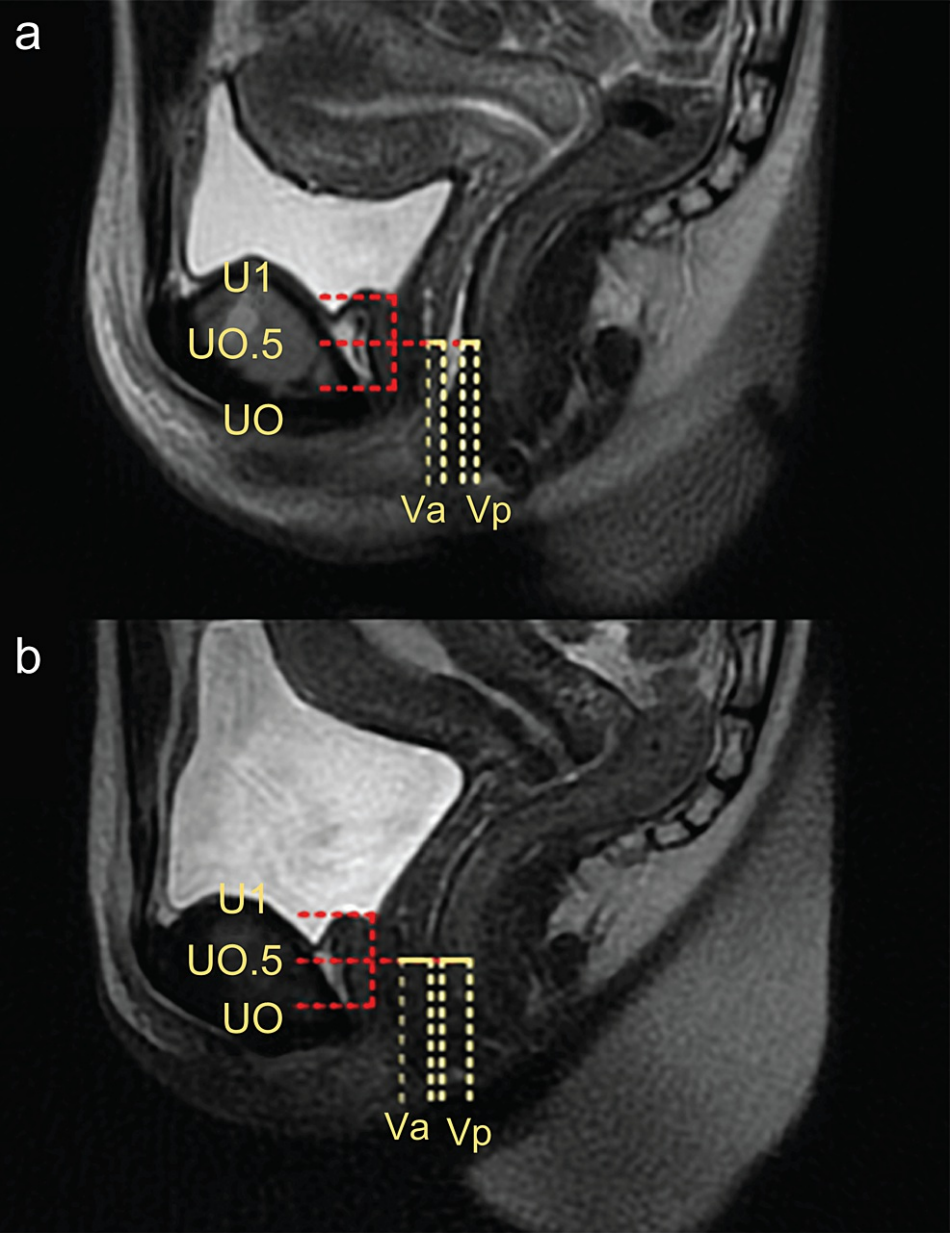
Vaginal wall thickness (a) Before treatment (T0), (b) after treatment (T3) U1: Upper end of the urethra (vesicourethral boundary), U0: Lower end of the urethra (urethral opening), U0.5: Midway between U0 and U1, Va: Thickness of the anterior vaginal wall at U0.5, Vp: Thickness of the posterior vaginal wall at U0.5

Figures 4a, 4b show vagina thickness at the height of the midpoint U0.5 between the upper extremity U1 (vesicourethral boundary) and the lower extremity U0 (urethral opening) of the urethra, with the anterior vaginal wall Va and posterior wall Vp increased. Compared with Va + Vp at T0, which was 8 mm, at T3, it increased to 12 mm, and lower vagina swelling may have suppressed the lower urethra.

**Figure 5:**
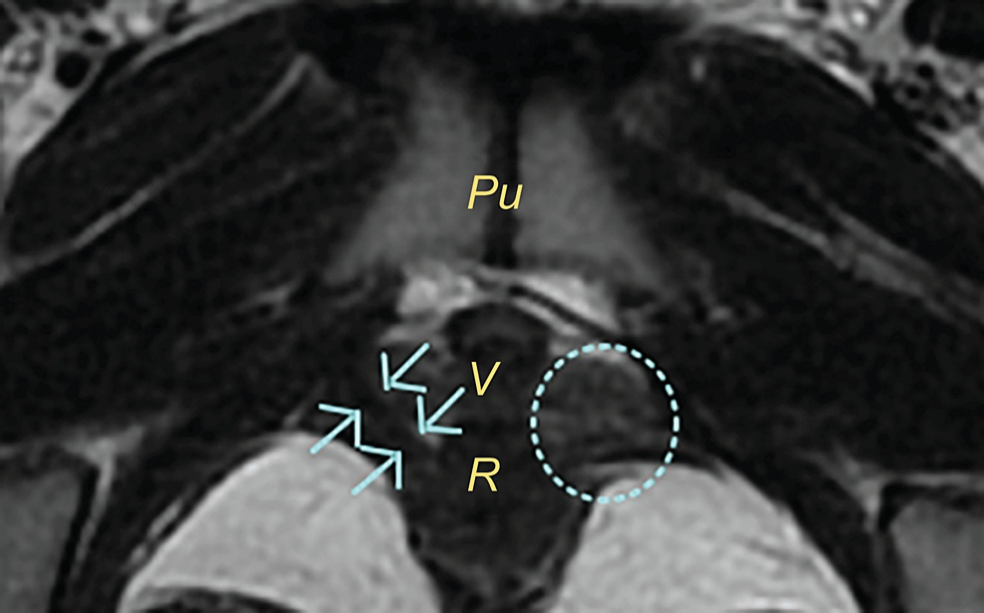
Axial view of the left anterior puborectalis muscle defect Blue arrow: Right anterior puborectalis muscle Blue dotted circle: Anterior defect of the left puborectalis muscle Pu, pubic bone; V, vagina; R, rectum

In Figure 5, the left puborectalis muscle anterior is missing and only the right puborectalis muscle is visible. This defect was the same at both T0 and T3.

Supplementary study

This study aims to investigate the anteroposterior urethral diameter and transverse urethral diameter on MRI. Female patients under 50 years without SUI who visited our hospital from April 2014 to March 2022 were included. The opt-out period was from April to October 2022. Among the patients whose urethra was measured by MRI under the same conditions as in the present study, we calculated the following criteria. MRIs were performed at Kanagawa Dental University, Yokosuka Kyousai Hospital, Uwamachi Hospital, and Sent-Yozefu Hospital.

Inclusion criteria were (1) previous vaginal delivery, (2) premenopausal, and (3) malignancy negative by urine cytology and uterine cytology. Exclusion criteria were (a) previous pelvic surgery, (b) uterine diseases such as uterine fibroids and endometriosis, and (c) urinary tract infection.

A total of 118 Asian women underwent MRIs. Five women were in exclusion criteria. (Undeniable urinary tract infection: two women, lack of confirmed urine cytology: one was, and without confirmed cervical cytology: two women). Finally, 113 were recruited. Recruited subjects are in a 95% confidence interval for the population.

Regression lines were calculated for each age group. An F-test for the commonality of the slope was performed between groups. P<0.05 was considered a significant difference. The statistical software R version 2.15.1 (R Core Team, Vienna, Austria) and Microsoft Excel version 1911 (Microsoft Corp., WA, USA) on a Windows 10 version 1903 (Microsoft Corp.) operating system was used for all analyses.

The characters of the eligible women are shown in Table 1. Ten women were over 20 years old and under 30 years old: the 20s women group. Forty-six women and 57 women were included 30 years old and under 40 years old: the 30s women group, 40 years old and under 50 years old: and the 40s women group, respectively. All women had an exercise routine of less than three days per week and had never been athletes.

**Table 1:** The demographics and populations of women

Number of samples	Total	113
	Over 20 years old and under 30 years old	10
	Over 30 years old and under 40 years old	46
	Over 40 years old and under 50 years old	57
Mean age		39.0±6.19 years old
Diabetes		3 (2.65%)
Smoking		9 (8.00%)
Hypertension		0 (0%)
Within one year after delivery		13 (11.5%)
Mean anteroposterior urethral diameter		13.21±2.0mm
Mean transverse urethral diameter		13.28±1.6mm

**Figure 6:**
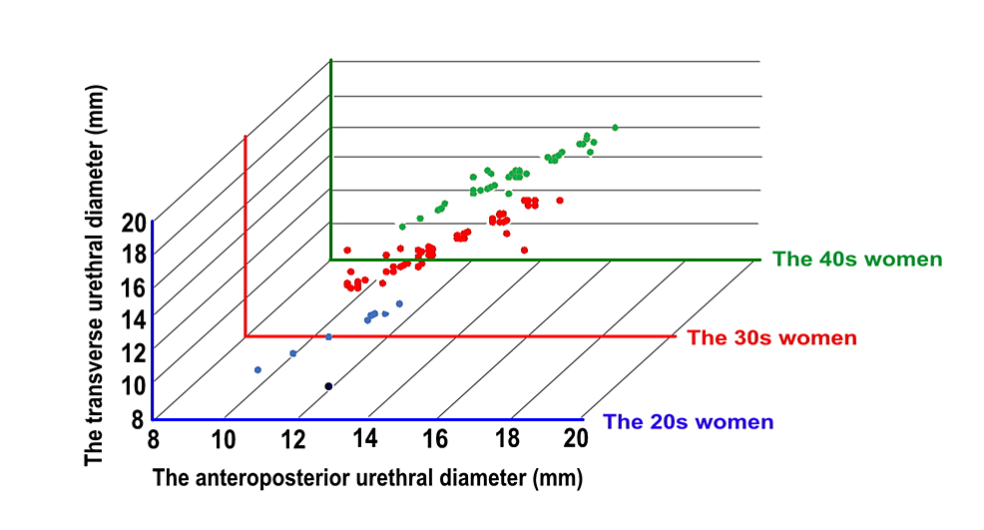
The anteroposterior urethral and transverse urethral diameters in the three age groups X line means the anteroposterior urethral diameter (mm), Y line means the transverse urethral diameter (mm), The blue dots and blue line show the 20s women group data, The red dots and red line show the 30s women group data, The green dots and green line show the 20s women group data, The black dot included in the blue X-Y lines is the EFA of this case report (T0)

Figure 6 shows a graph plotting the anteroposterior urethral diameter and transverse diameters of the urethra for each of the three age groups. In the 20s women group, the regression line is y=0.99x+0.09 (R^2^ = 0.995), indicating that the urethra is circular. In the 30s women and the 40s women groups, the regression lines are y=0.939x+0.72 (R^2^ = 0.931), and y=0.853x+2.02 (R^2^ = 0.868), respectively.

The analysis of covariance (ANCOVA) was performed for slope commonality of the three age groups. The slopes revealed no differences by age group (the 20s women group vs the 30s women group: p=0.626, the 30s women group vs the 40s women group: p=0.154, and the 20s women group vs the 40s women group: p=0.318).

Thus, premenopausal Asian women without SUI are predicted to have mean anteroposterior urethral diameter: 13.21±2.0mm and mean transverse urethral diameters: 13.28±1.6mm, which are circular regardless of age. The results are predicted to be circular regardless of age.

**Figure 7:**
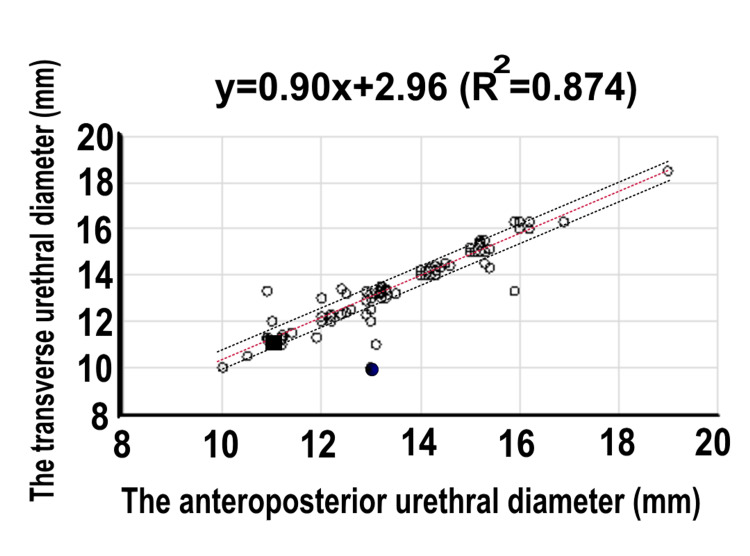
Regression line of the shape of the urethra in premenopausal women without SUI X line means the anteroposterior diameter (mm), Y line means the transverse diameter (mm), Open circle: premenopausal women without SUI, Closed circle: EFAs in this case report (T0), Closed square: EFAs in this case report (T3), Red dotted line: regression line, Black dotted line: Limits of 95% confidence interval

Figure 7 shows the anteroposterior and transverse diameters of the urethra in the entire sample of 113 individuals with open circle dots. The closed-circle dots are the EFAs discussed in this case report. This EFA is not within the overall 95% confidence interval (standard deviation: 0.600 mm). This means that the urethra of the pretreatment EFA is oval, which is different from the circular urethra of women without SUI.

## Discussion

To the best of our knowledge, there has never been a case report of an EFA who, after being unable to compete due to SUI, was able to return to a competitive life after treatment improved urinary incontinence. EFAs are known to experience SUI, but there are very few reports in which MRI was used to confirm the mechanisms of action and useful therapeutic targets. Furthermore, regarding VEL+UEL treatment effects, there have been no reports of anatomical changes and urinary incontinence on MRI over a 4-year long-term follow-up period. Hence, our research is of great significance from these three perspectives.

The natural history of SUI in EFAs is poorly understood [4]. The time of onset of SUI, in this case, was 17 years of age when the patient was nulliparous. Her SUI progressively worsened with time, and after child delivery, the patient often leaked all the urine in her bladder during a 400 m run. We found that even in the physically fit body of an EFA, urinary incontinence at a young age can be exacerbated by childbirth. Moreover, this patient experienced no improvement with PFMT and could not continue competing without some form of treatment. Therefore, therapeutic targets should be clarified in future cases.

Many studies have focused on both PFMT and PFM. First, we consider previous research papers on PFM exercises performed by this EFA. Lazaros et al. employed four sets of five tacklings and repeated them 20 times a day. This involves a series of diaphragmatic breathing and pelvic muscle relaxation exercises depending on the ability to palpate the vagina [18]. When implemented, 400 minutes are required as 1 minute each time. As in this EFA, the combination of resistance exercise and PFMT has been reported to be effective; Donelle et al. started with stretching and warm-up activities to promote flexibility and lower back movement in large muscle groups (hamstrings and quadriceps), followed by two to three sets of progressively loaded They reported a study of the PFMT that began with stretching and warm-up activities to promote flexibility of the large muscle groups (hamstrings and quads) and the addition of two to three sets of squats and deadlifts with progressive loads. The combination of strength training and PFMT is effective [19]. Based on these references, we predict that the PFMT conducted by this EFA would have been appropriate.

Many reports have studied PFM in EFA. Reports reviewing pelvic MRIs and translabial ultrasonographies lack a clear relationship between imaging findings and symptoms. Compared with non-athletes, EFAs have an increased cross-sectional area of the levator ani muscle (LAM), one of the PFMs. However, the levator hiatus area is large during the spontaneous Valsalva maneuver [20]. A study using the MOS and perineometer in nulliparous EFAs with a mean age of 24 years found that MOS and vaginal pressure in incontinent EFAs were 3.4 ± 0.7 and 33.9 ± 10.1 cmH_2_O, respectively. In contrast, EFAs without incontinence had a MOS of 3.2 ± 1.1 and vaginal pressure of 25.4 ± 12.8 cmH_2_O. For both, the number of EFAs with incontinence was higher [21]. Similarly, our case had adequate PFM contraction and a MOS of 4. This evidence suggests that lack of PFM strength in urethral support is one of the causes of urinary incontinence.

The evidence that PFMs, in this case, do not support the urethra stems from a left puborectalis muscle defect observed on MRI. This muscle defect may have been congenitally absent or failed to develop. The puborectalis muscle and the iliococcygeus and pubococcygeus, constitute the LAM [5]. A left puborectalis muscle defect indicates that LAM movement is unequal on the left and right sides. LAM injury leads to decreased urethral support which causes SUI [5,22]. However, even after successful treatment for SUI, the left puborectalis muscle is absent; therefore, treatment targeting LAM may not always be necessary.

The effects of VEL+UEL treatment may be explained by structural changes seen on MRI: the adipose spaces disappeared; the urethral cross-section, oval at T0, became round at T3; and increased vaginal wall thickness enabled urethral compression.

The first mechanism involves a space with fat. We reason that the probable cause of the existence of this space is overtraining since childhood. The muscles around the hip joint developed, and the pelvic bones grew and enlarged accordingly, but the urethra and vagina did not. Therefore, it can be inferred that SUI suddenly appeared at age 17. The disappearance of fatty spaces with VEL + UEL treatment may be related to the disappearance of the VAS, which interferes with sports. The patient explained that feelings of discomfort caused by pelvic organ shaking disappeared, making it easier to run short distances. This observation was not observed in previous studies on EFAs. Therefore, eliminating this additional fatty space may be a promising therapeutic target.

The second mechanism is that the cross-section of the urethra was not circular but oval. We believe that this meant that the urethra may have grown unevenly. From the present supplementary study (Table 1, Figures 6, 7), Asian women without SUI, premenopausal, have a circular urethra, as in past articles. Furthermore, it is observed that the urethra of this EFA is oval, different from theirs.

However, the lack of ISD at T0 indicates the presence of heterogeneous urethral striated muscle growth. Therefore, we believe that research on past EFAs has not received much attention. For UPP, MUP was 55 cmH_2_O, and MUCP was 54 cmH_2_O at T0. VEL + UEL treatment resulted in the MUP being 99 cmH_2_O and the MUCP being 78 cmH_2_O at T3. Furthermore, the roundness of the urethral cross-section on MRI indicated hypertrophy of the area. In other words, the muscle strength became uniform throughout the urethra.

There was no standardized normal or SUI reference value for UPP. A non-athlete with SUI reported that her MUCP was 61.0 (43.5-78.5) cmH_2_O [23]; however, MUCP in our case report was lower. Recent studies indicate that intra-abdominal pressure increases to levels exceeding the intraurethral pressure during high-motion sports [24]. Therefore, the patient, being a track and field athlete, experienced much intra-abdominal pressure.

This case was severe before treatment initiation. However, VEL+UEL treatment maintained an intraurethral pressure exceeding the normal value for Japanese patients, even three years after treatment. Hence, it has reached a level where incontinence can be controlled even when intra-abdominal pressures are high such as during competitions. Therefore, urethral morphology is an important therapeutic target.

Vaginal wall thickness is the third mechanism. From the pre-and post-treatment MRI, it can be said that the vagina was insufficient to support the urethra. Although there have been many reports of vaginal laser treatment, only a few studies confirmed changes in vaginal morphology after VEL treatment, and no reports confirmed this with MRI. A previous study reported that bladder neck mobility (BNM) from rest to strain is significantly reduced after VEL treatment [25]. BNM is considered a cause of SUI. The results indicate that one of the mechanisms of VEL's effect is the reduction of excessive BNM [25]. The same group used 3-D transperineal ultrasound to report reductions in the proximal, middle, and distal vaginal width and cross-sectional area in women with SUI after VEL treatment. Thus, the elasticity of the vagina itself increases to support the bladder neck, and urethra [26]. Reports have also shown that VEL treatment increases the epithelial thickness of the vaginal mucosa in patients with severe vaginal atrophy [10]. This evidence supports the theory that VEL treatment promotes vaginal collagen remodeling and increases vaginal thickness.

Vaginal wall thickness in this case also increased, which was the same result as the previous report. Thus, the lower vagina is an important therapeutic target for enhancing urethral support.

Our study is the first to report a VEL + UEL treatment. As mentioned above, there are many reports on VEL treatment, but only a few have shown morphological changes on imaging. Imaging studies are not yet available for UEL treatment of the human body. For patients with SUI, the ICIQ-SF showed a mean improvement of 64 % at three months and 40 % at six months with UEL treatment. Regarding the one-hour pad, leakage was reduced by 59 % at three months and 42 % at six months [13].

In MRI images showing the effect of the VEL + UEL treatment, the disappearance of the adipose layer in the pelvis cannot be explained only by vaginal wall thickening, as explained for VEL treatment. It is possible that the effects of both VEL and UEL established a system in which adipose tissue was burned. The rounded images of the urethra suggest UEL treatment effects. This urethral change is expected to be a similar laser treatment effect to studies showing that VEL treatment increases vagina elasticity [26]. Hence, increased vaginal wall thickness was considered an effect of VEL treatment, and an important finding is that this effect persisted for three years after treatment.

We discuss the interaction between the Smooth ErYAG laser and the vaginal mucosa and the depth of interaction between the Smooth ErYAG laser and the vaginal mucosa. It is important to understand how this interaction is happening. ErYAG laser emitting the light at 2,940 nm has an excellent absorption in water. The laser beam is absorbed very quickly at the first 5 µm of epithelium, where the light energy is converted to heat. Now, this heat is spreading through the tissue (due to the thermal diffusion and good thermal tissue conductivity), causing hyperthermia of the tissue. Using a very special Smooth modality, it is possible (by the train of non-ablative micro-pulses) to push this heat relatively deep into the mucosa - with well clinically proven and safe protocols (like is IncontiLase used in this study) consisting of a series of Smooth pulses delivered at one place, it is possible to push sufficiently high temperature (temp. window of 65°C to 45°C) to the depths of around 400-500 um, which means through the whole epithelium and well down to lamina priopria. In these mucosa layers, the hyperthermia produces collagen remodeling and initiates neocollagenesis and neoangiogenesis - the effects well proven with histological studies [9,27,28].

Aside from this hyperthermia effect, there is also an additional effect caused by fast (and high) thermal shocks produced by each or micro pulses inside the Smooth mode pulse. These fast shocks initiate the wound healing process, as tissue cells recognize them as wounds producing ones, although - being short enough, they do not produce any injuries. Effects of such wound healing process were detected even 2 mm deep in the mucosa [29,30].

Smooth mode ErYAG has been used in vaginal treatment for SUI and Genitourinary syndrome of menopause for more than 10 years [22,31]. It is expected to be applied to overactive bladder and interstitial cystitis [32,33]. Its thermal effects on the vaginal mucosa are well-proven in many clinical trials. Although it does not act directly on muscle or fat tissue, it is possible that this interaction may increase the number of cells in the respective tissues.

This research paper has two limitations. The first is that the relationship between SUI improvement and postpartum recovery is not clear. There is currently no way to know if this EFA would have improved SUI if the PFMT had continued over time without laser treatment. Yang et al. performed pelvic floor ultrasonography in 26 postpartum Asian women at 36 weeks of gestation and two, four, six, and 12 weeks postpartum. They focused on bladder neck descent (BND). BND can be considered the cause of SUI. BND was lowest at 36 weeks' gestation and increased until six weeks postpartum. The highest BND value was observed at six weeks postpartum and was significantly higher than at 36 weeks' gestation. They reported that her BND values at 12 weeks postpartum were significantly lower than those at six weeks postpartum [34]. Hilde et al. compared a group that received PFMT daily at home for 16 weeks with a group that did not. At six months postpartum, the number of women with complete levator ani (LA) muscle avulsion decreased from 27 to 14 in the PFMT group (44% reduction) and from 28 to 17 in the control group (39% reduction). They reported that the difference between groups was not significant [35]. Most studies of postpartum urinary incontinence are for months 3 to 6; few studies are available for months 6 to 12 [36]. The EFA in this case report did not improve at all 12 months after giving birth, and it is possible that it would not have improved without the laser treatment. In addition, this EFA had SUI since under 18 years old. A prospective randomized trial is needed to prove the mechanism of this study.

Our next limitation is the lack of research on changes in the size and shape of the urethra and urinary incontinence. In this study, SUI is evaluated beforehand using pad testing, urodynamic studies, and UPP. This study found that the MRI information was very informative, as was the information from these SUI assessments. Supplemental studies indicate that the urethra is circular in Asian women, consistent with previous reports. From these studies and data, it is noted that the urethra of this EFA is oval. A cross-sectional study comparing athletes and non-athletes is needed to determine if this characteristic is true for all athletes with SUI.

## Conclusions

Herein, we reported a case of an EFA for which VEL + UEL treatment for severe SUI was effective for up to three years. This treatment improved the patient’s competition results as well as urinary incontinence. The conclusion may offer practitioners a new combined VEL + UEL treatment alternative. Analysis by MRI confirmed the absence of the left puborectalis muscle, uneven growth of the urethra, thin vaginal walls, and space for the pelvic adipose layer before treatment. MRI before and after treatment confirmed that the urethra, vagina, and fat layer had improved. These new findings will be useful for mechanistic studies of VEL + UEL treatment.
